# Operating room nurses’ lived experiences of ethical codes: A phenomenological study in Iran

**DOI:** 10.1016/j.ijnss.2021.05.012

**Published:** 2021-06-04

**Authors:** Fateme Aghamohammadi, Behzad Imani, Mahnaz Moghadari Koosha

**Affiliations:** a Department of Operating Room, Student Research Committee, Hamadan University of Medical Sciences, Hamadan, Iran; b Department of Operating Room, School of Paramedicine, Hamadan University of Medical Sciences, Hamadan, Iran

**Keywords:** Ethical codes, Qualitative study, Phenomenology, Iran, Operating room nursing, Perioperative nursing

## Abstract

**Objective:**

Operating room nurses, as essential members of health care teams, often face ethical challenges in the operating room. By using the ethical experiences of operating room nurses, a better understanding of ethics in the operating room can be achieved, which can lead to better nursing decisions in the face of these challenges. Therefore, this study was conducted to investigate operating room nurses’ lived experiences of ethical codes.

**Methods:**

A hermeneutic phenomenological study was performed in Hamadan (Iran) from February 2019 to November 2020. Ten operating room nurses were selected as participants by purposive sampling. Data were collected through in-depth and semi-structured interviews. Data analysis was performed based on Van Manen methodology.

**Results:**

Data analysis revealed three main themes and 11 sub-themes representing the operating room nurses experience of the ethical code. The main themes were; adherence to professional commitments, preserving patient dignity, and respect to colleagues.

**Conclusion:**

The results underlined ethics and ethical values in the operating room. Due to the intense interactions between operating room nurses with the patient and surgical team, commitment to ethics by nurses can lead to improving quality of care and interactions among members of the surgical team. It is suggested that using these codes as a guideline and a framework could be developed to improve the ethical and professional performance of operating room nurses.

## What is known?

• The operating room is a stressful work environment and full of ethical challenges for nurses. Although many studies have focused on ethics in general nursing, there has been limited assessments of operating room nurses experiences of ethical values

## What is new?

• Given the many ethical challenges for nurses in the operating room, using these codes in interaction with patients and colleagues can improve the quality of interactions and patient care in the operating room. Healthcare authorities and hospital managers can use these codes to design a framework for the ethical practice of operating room nurses

## 1 Backgrounds

The operating room is a high-tech and stressful work environment in which the patients are exposed to invasive procedures. Since in the operating room, a wide range of healthcare professionals perform their tasks with overlapping, interpersonal conflicts occur frequently. Often, life-threatening situations and events requiring immediate decision-making happen in this environment. Because of these characteristics, operating room nurses are exposed to ethical challenges [1,2].

Ethical challenges are complex issues that are not easily resolved. These problems could be new issues, daily issues, or situations that have to be decided among several options [3,4]. The most common ethical challenges in operating rooms are: not sincerely communicating with patients, ignoring patients’ expectations, failure to follow the sterility principles, wrong surgery, refusing of admitting some patients for surgery, failure to receive informed consent from the patients, and incorrect registration of materials used [1].

Blomberg and Bisholt (2018) found that the responsibility of operating room nurses contains two aspects. The first is the responsibility to have professional skills and knowledge in preparation of patient and equipment needed for various surgeries. The second is the moral responsibility of operating room nurses in having a professional and respectful relationship with patients and colleagues [5].

Also, Bilik and Kaya (2017) stated that the ethical responsibility of operating room nurses in this environment full of moral challenges is increasing. Prevention of these challenges requires the attention and planning of nursing managers and trainers in order to educate professional ethics in the operating room and develop the moral sensitivity of operating room nurses [1,6].

Ethical challenges can lead to psychological and physical symptoms, reduced job satisfaction, and even improper or inadequate nursing care [7]. Unfortunately, despite being preventable, most of the immoral behaviors reported in health care occur in operating rooms [8]. For this reason, operating room nurses must not only continually improve their professional skills but also their moral competence [9].

Observance of professional ethics creates a sense of trust in the nursing profession, sense of security and peace in patients, reduces the patients’ hospitalization time, creates proper interactions among nurse-patient and nurse-colleagues, and finally reduces the hospitalization costs [10]. Thus, it is appropriate for nurses in different positions to provide services based on common values, which are usually reflected in the ethical codes of nursing [11,12].

Nursing ethics codes define the values and standards of professional behavior and are a guideline for nurses’ ethical responsibilities and ethical decisions [7,13]. Stievano and Tschudin (2019) stated that in many countries, there are no ethical codes for some nursing specialties, such as operating rooms [14]. In the absence of a written code of ethics, nurses could be confused about ethical issues and do not know exactly what decision to make. Patients also do not know what to expect from nurses, which leads to anxiety and decreased satisfaction [11].

Due to the sensitivity and complexity of working conditions in the operating room and a limited number of studies conducted in professional ethics in the operating room in Iran, the present study was conducted to investigate the lived experience of operating room nurses of ethical codes.

## 2 Methods

### 2.1 Study setting and sampling

This qualitative study was conducted based on Heidegger’ s philosophy and hermeneutic phenomenology in Hamadan (Iran) from February 2019 to November 2020. Hermeneutics is a systematic approach for phenomenon study from an interpretive perspective view which gains a deeper understanding of lived experiences [15]. In this study, we collected the nurses with rich experience of subject and ability to express and desire to participate in this research. Inclusion criteria were at least 1 year of work experience in operating room and desire to participate in the study. Also, the exclusion criteria were dissatisfaction to participate in the study. Ten operating room nurses were purposefully selected. In qualitative studies, the sample size should be based on information needs. Hence, a guiding principle of sample size is data saturation, and sampling is performed since no new information is obtained [16]. Patients’ consent to participate in the present study was first obtained orally and then in writing. In our study, sampling was continued to achieve data saturation by performing 10 interviews. To obtain maximum diversity, the participants were selected from the operating rooms of several hospitals affiliated with Hamadan University of Medical Sciences. As shown in Table 1, 4 women and 6 men participated in the study, of which 6 were operating room nurses, 3 were anesthesia nurses and 1 was operating room manager. Their work experience was 2–21 years, 2 of participants had master’s (MSc), and 8 of them had bachelor’s (BSc) academic degrees in nursing.

**Table 1:** Descriptive characteristics of participants.

Participants	Age (years)	Gender	Education level	Specialty	Working experience (years)
P1	32	Female	BSc	OR nurse	9
P2	40	Female	BSc	OR nurse	15
P3	36	Male	BSc	OR nurse	12
P4	29	Male	MSc	OR nurse	5
P5	50	Female	BSc	Anesthetic nurse	21
P6	42	Male	BSc	OR nurse	18
P7	26	Female	BSc	Anesthetic nurse	6
P8	43	Male	MSc	Anesthetic nurse	14
P9	25	Male	BSc	OR nurse	2
P10	37	Male	BSc	OR manager	15

*Note:* BSc = Bachelor of Science. MSc = Master of Science. OR = operating room.

### 2.2 Data collection

Data were collected using semi-structured, face-to-face, in-depth interviews. Ten interviews were conducted with the participants by the first author. Nine interviews were conducted in the nurses’ restroom, and one interview was undertaken in the operating room manager’s office. The mean duration of interviews was 50 min (40–70 min). Each participant was interviewed once because the acquired information was complete, clear, and sufficiently detailed. Before the start of the interview, the goals of the study were stated, and the participants were given informed consent to record the interviews. The interviews initiated with the question, such as “What is your experience with the observance of ethical principles in the operating room?” and “What is the first thing that comes to your mind when the term ethical principles in the operating room are mentioned?” In order to make the interview clearer and deeper, the exploratory questions, such as “Explain more? And what do you mean?” were asked. The recorded interviews were implemented verbatim in less than 24 h by the first author, and the transcripts of the interviews were referred to the participants, and they were asked to approve it. Thus, in the case of ambiguity, the researcher could be re-followed**.**

### 2.3 Data analysis

Immediately after the first interview and simultaneous data collection, data were analyzed using Van Manen’s (1997) approach [15] by authors. Six steps of this approach were as follows; 1) turning to the nature of lived experience and paying attention to this, 2) obtaining descriptions about the lived experience through investigation of the phenomenon as it is lived, not as it is conceptualized, 3) engagement in thematic analysis through reflecting the essential themes which characterize the phenomenon, 4) engagement in phenomenological writing to describe the phenomenon via the art of writing and rewriting, 5) establishment and maintenance a strong and oriented relation to the phenomenon, and 6) creation of coherence and context balancing by considering the parts and the whole.

### 2.4 Ethics approval

This study was approved by the Research Council and Ethics Committee of Hamadan University of Medical Science (IR.UMSHA.REC.1398.404) and Proposal number No: 9805223881. Written informed consent was obtained from the participants in the study. Maintaining the anonymity, confidentiality of information, and the right to withdrawal from the study were also considered. The time and place of the interview were also arranged according to the coordination and request of participants.

### 2.5 Trustworthiness

In order to ensure the rigor of the study, the following four main criteria were employed; credibility, confirmability, dependability, and transferability [17]. To increase credibility, the researchers conducted member checks with participants during the process of data collection and analysis. In each stage, data or results were returned to participants to check the accuracy and clarity of their experiences, and some changes were applied if needed. Moreover, factors like long-term and ongoing engagement with data and diversity of participants in terms of demographic characteristics (e.g. gender, age, and work experience) supported the credibility of the findings. Dependability referred to the stability of data over time and conditions [16]. An audit trail, themes, sub-themes, and all evidence and documents were used to maintain participants’ experiences and improve dependability. To establish confirmability, the research team’s collective opinions were included in all stages of data analysis. Furthermore, all the study steps were recorded with details. Transferability of the findings was maintained through maximum variation sampling [18].

## 3 Findings

A total of 10 operating room nurses (4 women and 6 men) with a mean work experience of 11.7 years participated in the present study (Table 1). Analysis of the interviews resulted in the finding of 530 primary codes. In data analysis, following deletion of duplicate codes and merging similar items. Finally, three main themes and 11 sub-themes were extracted (Table 2), as follows: adherence to professional commitments, preserving patient dignity, and respect to colleagues.

**Table 2:** Operating room nurses’ lived experiences of ethical codes: themes and sub-themes.

Themes	Sub-themes
Adherence to professional commitments	•Promote professional and personal competence •Commitment to honesty •Commitment to justice •Punctuality •Responsibility •Observing the correct principles of sterilization and aseptic
Preserving patient dignity	•Respect for patient pPrivacy •Emotional and psychological support of patients •Respect for the patient autonomy
Respect to colleagues	• Respectful relationship with surgical team •Owning a teamwork spirit in the operating room

### 3.1 Adherence to professional commitments

Based on participant’s statements, many human resources with different specialties are working together in operating rooms with the goal of optimal care provision for the patients admitted to the operating room for various reasons, often due to emergencies. Thus, operating room nurses face various issues and challenges in their decisions and interactions with colleagues and patients. They also stated that, regardless of the nature of problems and deficiencies of the workplace, nursing care should benefit patients and prevent harm to patients. Thus, it is the moral duty of operating room nurses to constantly consider ethical principles and standards in providing physical and psychological care to these patients. According to the findings of this study, adherence to professional commitments can guide nurses in a large part of these ethical problems. This theme has six sub-themes which are as follows.

#### 3.1.1 Promote professional and personal competence

The participants also believed that the nature of patient care in the operating room is complex and occurs in a unique environment. Thus, operating room nurses should constantly improve their scientific and practical information and skills to provide principled and ethical care for the patient. In this regard, a participant with 2 years of work experience stated, *“Honestly, once, I wanted to work with the C-ARM, but I did not know. It made the surgeon angry, the operation lasted longer, and once or twice I exposed high-dose radiation to the patient. After that day, I tried to have information for the smallest device in the operating room, and I also participated in training courses that are held so that I am always updated.”* (P9)

#### 3.1.2 Commitment to honesty

In the surgical team, nurses and physicians are working to achieve a single goal, which is to restore or improve patients’ health. In order to gain the trust and satisfaction of patients and colleagues, it is necessary always to have honesty among the members of the surgical team and also in dealing with patients and their families. One of the anesthetic nurses with 21 years of work experience stated that, *“Once I injected medicine for a patient without an anesthesiologist*’*s prescription, which caused a severe drop in the patient*’*s blood pressure. I was much stressed. At first, I was afraid that if I told the anesthesiologist, I would get into trouble. But since the patient might have a cardiac arrest or a problem, I quickly told the truth. Thank God nothing bad happened to the patient.”* (P5)

#### 3.1.3 Commitment to justice

In the operating room, nurses work with different thoughts and ideas, and usually, different patients (in terms of religious beliefs, social status, economic status, language, etc.) are brought to the operating room for surgery. Therefore, most nurses believe that these issues may lead to prejudice and unfair distribution of care resources. They stated that, according to the above-mentioned conditions, and since the patient’s companions are not present in the operating room to monitor how care is provided to the patient, it is the duty of a morally committed nurse to treat all patients fairly without discrimination. A contributor with 9 years of work experience said, *“I remember we had a patient here for surgery who came from prison. Unfortunately, our anesthesia nurse did not treat the patient well. He did not change the tubes of the anesthesia machine and the mask to be sterile because the patient was an imprisoned person. However, in another shift, when we worked together and the patient was one of the officials of the hospital, he worked very carefully and was extremely respectful and polite.”* (P1)

#### 3.1.4 Punctuality

According to the experiences stated by nurses, health professionals are highly interdependent and work under time pressure in surgical teams. The timely presence of the surgical team and patient in the operating room is an ethical issue because both groups are mutually respected, and it prevents the patient from worry, anxious, and wasting time. The operating room manager with 15 years of working experience stated, *“I have seen many times that the elective patients are picked up from the ward much earlier than the time of their surgery, and the patient stays in reception for a long time. Personnel traffic, the presence of various patients and waiting are annoying for the patients, making them agitated, and the blood pressure rises. I warned the colleagues about this and told them that whenever the room was ready, and the surgeon was present, the patient should be called to the operating room so that the patient can experience the shortest time between his presence in the operating room and anesthesia.”* (P10)

#### 3.1.5 Responsibility

Most nurses agreed with the definition of responsibility, meaning a sense of commitment to conducting the right job description. They stated that providing quality care is the right of all patients and the responsibility of all nurses. Absence or deficiency in this field causes patients’ distrust of nurses, worries, and harms patients. In this regard, one of operating room nurses with 5 years of working experience stated, *“Once the circular nurse in our room gave the sample of a patient to a student for registration. Unfortunately, the student did not do this correctly, and the patient’s sample was lost, and the purpose of that surgery was only to take a sample. Well, it was the responsibility of the circular nurse to register the sample. However, he failed and did not take it himself.”* (P4)

#### 3.1.6 Observing the correct principles of sterilization and aseptic

Based on the experiences expressed by operating room nurses, site infection is an important complication after surgery which sometimes causes patients to be referred back to the operating room for re-surgery or long-term hospitalization of patients after surgery. Operating room nurses can play an important role in preventing and controlling this complication by properly observing the principles of aseptic and sterile regarding hand scrub, equipment used in surgery, skin preparation (prep), and surgical drape. One of the interviewees with 6 years of working experience stated, *“Once, we had a laparotomy, and the scrub nurse set the operating table, and everyone was ready to start the surgery. At one point, the scrub nurse said that the set label was not sterile. He ordered everyone to scrub their hands again and change their surgical Gown and gloves; he did it himself and also put all those tools aside. He then asked me, as a circular nurse, to quickly bring other sterile equipment for surgery.”* (P7)

### 3.2 Preserving patient dignity

Respect for human dignity is one of the most important rights of all human beings, especially patients. It is also essential to maintain patients’ dignity to provide quality health care. Nursing ethics requires that nurses respect patient privacy, treat and support them appropriately, and respect patients’ wishes and decisions. This theme has three sub-themes which are as follows.

#### 3.2.1 Respect for patient privacy

Participants stated that we meet patients in the operating room who are extremely vulnerable, as their usual privacy is lost in this environment and the patient is unconscious. They delegate their authority to the surgical team and literally put their lives in our hands, thus their reputation may be compromised. Accordingly, the protection of patient privacy is a major concern for all health professionals, especially operating room nurses who are involved in patient care. A nurse with 9 years of work experience in operating room commented on patient privacy and stated, *“In genital surgery of a woman we had a few days ago, I felt the patient very uncomfortable because of the presence of male students in the room. So I asked them to go out of the room and close the door so that the patient would be comfortable.”* (P1)

#### 3.2.2 Emotional and psychological support of patients

The participants believed that the presence of patients in the operating room is always associated with stress and many worries and questions. All these factors also affect the anesthesia and surgery process. It is the duty of an ethically committed nurse to understand the patient’s concerns and be kind to the patient and respond to their needs. Regarding the emotional and psychological support of patients, a participant with 14 years of experience said, *“Once our patient was a sick child, about 12 years old, when we saw him he hides under the blanket, and his hand was shaking. I saw our colleague sitting next to him, holding his hand, caressing him, talking to him, and making him talk about his fear and worries about being in the operating room. Finally, before the anesthesia, the patient became very calm and relaxed.”* (P8)

#### 3.2.3 Respect for patient autonomy

Participants also stated that in the operating room, as in other wards of the hospital, the nurses and physicians should be committed to respect patients’ decisions and their right to choose treatment methods, surgical and anesthesia methods as far as is beneficial to the patient. Another participant with 5 years of experience stated, *“We had a patient who previously undergone laparotomy surgery and her skin sutured with a stapler.* Because of this, the patient was very upset *and had many scars left. This time, when she came to the operating room, she asked us to suture her skin with nylon. At the end of the operation, I emphasized to the surgeon that he should suture her skin with nylon according to her request.”* (P4)

### 3.3 Respect to colleagues

The operating room is an environment with many challenges and stresses. Operating room nurses stated that all colleagues, like patients, should be respected. Because they believed that the quality of surgical procedures and patient safety and the nurses’ scientific skills, it depends on their ethical skills and effective communication with members of the surgical team.

#### 3.3.1 Respectful relationship with colleagues

Participants also stated that respect for colleagues’ privacy, their position and personality, existing differences, and knowledge sharing with other colleagues would enhance the sense of empathy and collaboration skills and ultimately create a sense of calm in the workplace and provide optimal care to patients. A nurse with 18 years of working experience stated, *“Personally, I feel useful and motivate when my co-workers, who are younger and had less work experienced than me, pay attention to my experiences and opinions; I do my duties in the operating room with a better spirit and more motivation.”* (P6)

#### 3.3.2 Owning a teamwork spirit in the operating room

The basis of work in the operating room is teamwork. The surgical team consists of different professions with different tasks which work together to achieve a common goal, understand the complexity of the clinical situation, make appropriate decisions, and perform safe surgery. The performance of all these members has a direct effect on outcomes of surgical work. From the participants’ perspective, owning a spirit of cooperation in the operating room was crucial. One of them with 3 years of work experience reported, *“Once we had brain tumor surgery that* *was too long. In the middle of the operation, I was not feeling well, and I did not tell the circular nurse. But he quickly noticed that I was tired and in a bad mood and went to the operating room manager*’*s office to ask for another nurse to help us. One of the co-workers who were in the manager*’*s office came quickly and voluntarily instead of me, I was able to go and get some rest. I really thank them. I was in a bad mood and I could have made a mistake because of this fatigue*.*”* (P3)

## 4 Discussion

In this study, the nurses’ experiences regarding the ethical codes in the operating room were examined based on Heidegger’s philosophy and hermeneutic phenomenology. Semi-structured interviews were performed, and data analysis revealed three main themes; adherence to professional commitments, preserving patient dignity, and respect for colleagues.

Ethical codes are a guide to help make better interactions among nurse-patient and nurse-colleagues and help make ethical decisions in complex professional situations. Compiling codes using the experiences of operating room nurses can lead to better cooperation in their acceptance and application [11].

The primary main theme of this study was adherence to professional commitments. The sub-themes include promoting professional and personal competence, commitment to honesty, commitment to justice, punctuality, accountability, and observing the correct principles of sterilization and aseptic principles.

In line with adherence to professional commitments, Bakhtiari et al. (2020) stated that the promotion of professional commitments and striving to improve is one of the main themes of ethical behaviors in operating room [2]. Barać et al. (2018) also stated that providing professional services by nurses without a sense of professional commitment is difficult with a negative impact on the quality of care [19]. According to Halpern & Spandorfer (2014), professional commitments include professional competence, be honest with the patient, keep secrets, proper interaction with the patient, equitable distribution of limited resources, scientific knowledge, maintaining trust and professional responsibilities [20].

Also important ethical factors listed in the International Council of Nurses (ICN) Code of Ethics for Nurses 2012 include honesty, kindness, generosity, equality, fairness, justice, and courage [14]. Having up-to-date professional knowledge, accepting responsibility for performing tasks properly and maintaining patient safety, humility in dealing with patients, respect for patients’ right to make decisions are examples of ethical responsibilities of operating room nurses [5]. Sterile technique is an essential principle of patient safety which reduces the risk of microbial transmission to patients during surgery. Adherence to aseptic principles and patient safety concerns in the operating room are considered as responsibility of surgical team members, including nurses, surgeons, and anesthesiologists [21]. All surgical team members, including operating room nurses, must be committed to the proper observance of sterile principles [22]. Regarding the importance of punctuality, it can be stated that delays in the operating room processes have a negative effect on both patients and health care staff [23]. Thus, Starting surgery at the arranged time is a respectful attitude towards the patient and colleagues [24].

The second main theme was preserving patient dignity with the following sub-themes: respect for patient privacy, emotional and psychological support of patients and respect for patient autonomy.

In this line, Šaňáková & Čáp (2019) stated that dignity is one of the most important values that patients perceive in nursing care [25]. Respect for human dignity is in line with the international ethical codes of nursing [26]. Paying attention to patients’ dignity strengthens their self-esteem, faster recovery of patients, prevents stress and negative consequences in them [27].

Hanssen et al. (2020) also stated that respecting and patients care, creating a sense of calm and confidence in patients, respecting members of the surgical team by accepting their responsibilities and respecting the duties of others, and communicating with other team members are key ethical skills for operating room nurses [28]. In this regard, Maluwa et al. (2018) mentioned some of the criteria of nurses’ moral competence; kindness, compassion, responsibility, discipline, accountability, communication, honesty, and respect for values, dignity, and human rights [29]. The American Nurses Association (ANA) Code of Ethics stated that the ethical care of patients in the operating room requires that operating room nurses follow the safe and legal guidelines and consider themselves obliged to support patients [28]. Medical ethics requires physicians who must be polite to patients, maintain patients’ privacy, and respect their beliefs and values [30]. Privacy is essential to create a sense of security in patients and physical and psychological support of patients [31]. In nursing and health care, patients have the right to make decisions independently, and respect for patients’ independence in health care has recently received special attention [32].

The third main theme found through analyzing the lived experience of nurses was the presence of respectful relationships with colleagues, including the following sub-themes: a respectful relationship with the surgical team and owning teamwork spirit in the operating room.

The results of a study conducted by Navalta, Stone, and Lyons (2019) showed that colleagues, regardless of age, race, religious or political views, gender, or sexual orientation, should be respected for their professional work [33]. Collaboration between different professions is essential to provide high-quality care and safe surgery [34]. Hussen et al. (2020) also stated that lack of respect and teamwork in the operating room leads to endangering the patient’s safety and has negative effects on the performance of surgical team members. Therefore, respecting colleagues and participating in the creation of a calm and safe caring environment is one of the ethical skills of operating room nurses [28]. In particular, humility and respect for colleagues in all disciplines are essential [35]. Good communication and cooperation in the operating room leads to a calm working environment for staff and reduces medical errors and complications for patients. Thus teamwork in the operating room is necessary to ensure the outcome of treatment [36]. Performing safe surgery for patients is based on constructive communication among members of the surgical team and teamwork [37]. Thus, to provide the best surgical care for the patient, all team members must work together, communicate well, and coordinate [38]. The mentioned studies stated that respecting colleagues and owning a team spirit in the operating room are ethical skills.

The purpose of designing these ethical codes was to provide a guideline and a model for the professional performance of operating room nurses. It is necessary to internalize these values in practice and encourage the implementation of these values in nursing care in the operating room. As much as possible, an attempt was made to provide a clear and practical set in the daily activities of nurses by addressing different areas of work in the operating room. These ethical codes might be used by operating room nursing managers as a guide to improving the performance of operating room nurses.

*Limitations* The study findings are a reflection of the experiences of a small group of operating room nurses. Thus, the findings should be generalized with caution. To alleviate this limitation, nurses were selected from the operating rooms of different hospitals affiliated to Hamadan University of Medical Sciences and with the variety as much as possible. One participant also refused to continue the interview, which was managed by replacing another participant.

## 5 Conclusion

The findings of this study demonstrated the operating room nurses’ experiences of ethical behavior in the operating room. Concepts such as adherence to professional commitments, preserving patient dignity, respect for colleagues were crucial for participants. By identifying the ethical codes and adherence of nurses, the quality of nurses’ interactions with patients and colleagues is improved, leading to improved quality of patient care.

*Implication for nursing management* The results of this study could be provided to the University Ethics Committee and hospital operating room managers. It is suggested that under the supervision of ethics experts, a guideline and a framework could be developed to improve the ethical and professional performance of operating room nurses by the use of these codes. The results of this study can also be used in the education of nursing students. In addition to acquiring scientific skills, they need to develop and strengthen knowledge and moral attitude. Further, the researchers are needed to explore the experiences of other health professionals in the field of ethical codes. It is hoped that by application the results of this study, researchers can take an effective step towards improving patients’ rights and safety in operating room.

## Funding

The study was funded by Vice-chancellor for 10.13039/501100004697Research and Technology, Hamadan University of Medical Sciences (No. 9805223881).

## CRediT authorship contribution statement

**Fateme Aghamohammadi:** Conceptualization, Methodology, Software, Formal analysis, Investigation, Resources, Data curation, Writing – original draft, Project administration, Funding acquisition. **Behzad Imani:** Conceptualization, Methodology, Software, Validation, Formal analysis, Investigation, Data curation, Writing – original draft, Writing - review & editing, Visualization, Funding acquisition. **Mahnaz Moghadari Koosha:** Conceptualization, Methodology, Formal analysis, Investigation.

## Declaration of competing interest

The authors declare that they have no competing interests.
