# Supplementary material for: Operating room nurses’ lived experiences of ethical codes: A phenomenological study in Iran
Source: Int J Nurs Sci. 2021 Jun 4;8(3):332–8. doi: 10.1016/j.ijnss.2021.05.012 (PMC8283715; doi:10.1016/j.ijnss.2021.05.012)
Supplement: Multimedia component 1 [file mmc1.docx]

伊朗手术室护士关于手术室护理伦理的真实体验

Fateme Aghamohammadi, Behzad Imani, Mahnaz Moghadari Koosha

【摘要】

目的 手术室护士作为医疗队伍的重要成员，在手术室中经常面临伦理挑战。通过了解对手术室护士关于伦理的真实体验，可以更好地理解手术室的伦理原则，从而在面对这些挑战时做出更好的护理决策。本研究旨在探讨手术室护士关于伦理规范的真实体验。

方法 采用目的抽样法， 2019年2月至2020年11月在伊朗哈马丹市选取10名手术室护士作为研究对象，进行现象学解释学研究。通过半结构化访谈收集资料，并采用Van Manen方法进行资料分析。

结果 资料分析呈现出3个主要主题和11个子主题。手术室护士伦理原则的3个主要主题为：坚持专业承诺、维护患者尊严和尊重同事。

结论 研究结果强调了手术室的伦理和伦理价值。由于手术室护士与患者和手术团队成员之间需密切互动，护士对伦理规范的承诺可以改善医疗护理质量和手术团队成员之间的交流。建议将这些伦理原则作为指导方针和框架加以发展，以提高手术室护士的职业道德和专业水平。

【关键词】伦理准则； 伊朗； 道德；手术室；手术室护理；围手术期护理

通信作者：Behzad Imani, E-mail: behzadiman@yahoo.com
